# Articular inflammation induced by an enzymatically-inactive Lys49 phospholipase A_2_: activation of endogenous phospholipases contributes to the pronociceptive effect

**DOI:** 10.1186/s40409-017-0104-0

**Published:** 2017-03-23

**Authors:** Renata Gonçalves Dias, Sandra Coccuzzo Sampaio, Morena Brazil Sant’Anna, Fernando Queiroz Cunha, José María Gutiérrez, Bruno Lomonte, Yara Cury, Gisele Picolo

**Affiliations:** 1 Special Laboratory of Pain and Signaling, Butantan Institute, Av. Vital Brazil, 1500, São Paulo, SP CEP 05503-900 Brazil; 2 Laboratory of Pathophysiology, Butantan Institute, São Paulo, SP Brazil; 3 Department of Pharmacology, Institute of Biomedical Sciences, University of São Paulo, São Paulo, SP Brazil; 4 Department of Pharmacology, Faculty of Medicine of Ribeirão Preto, University of São Paulo, Ribeirão Preto, SP Brazil; 5 Clodomiro Picado Institute, Faculty of Microbiology, University of Costa Rica, San José, Costa Rica; 6 Healthy Sciences Institute, Paulista University (UNIP), São Paulo, SP Brazil

**Keywords:** Lys49-PLA_2_, Myotoxin II, Arthritis, *Bothrops asper*, Phospholipase

## Abstract

**Background:**

Arthritis is a set of inflammatory conditions that induce aching, stiffness, swelling, pain and may cause functional disability with severe consequences to the patient’s lives. These are multi-mediated pathologies that cannot be effectively protected and/or treated. Therefore, the aim of this study was to establish a new model of acute arthritis, using a Lys49-PLA_2_ (*Bothrops asper* myotoxin II; MT-II) to induce articular inflammation.

**Methods:**

The articular inflammation was induced by MT-II (10 μg/joint) injection into the left tibio-tarsal or femoral-tibial**-**patellar joints. Cellular influx was evaluated counting total and differential cells that migrated to the joint. The plasma extravasation was determined using Evans blue dye. The edematogenic response was evaluated measuring the joint thickness using a caliper. The articular hypernociception was determined by a dorsal flexion of the tibio-tarsal joint using an electronic pressure-meter test. The mediators involved in the articular hypernociception were evaluated using receptor antagonists and enzymatic inhibitors.

**Results:**

Plasma extravasation in the knee joints was observed 5 and 15 min after MT-II (10 μg/joint) injection. MT-II also induced a polymorphonuclear cell influx into the femoral-tibial-patellar joints observed 8 h after its injection, a period that coincided with the peak of the hyperalgesic effect. Hyperalgesia was inhibited by the pretreatment of the animals with cyclooxygenase inhibitor indomethacin, with type-2 cyclooxygenase inhibitor celecoxib, with AACOCF_3_ and PACOCF_3,_ inhibitors of cytosolic and Ca^2+^-independent PLA_2_s, respectively, with bradykinin B_2_ receptor antagonist HOE 140, with antibodies against TNFα, IL-1β, IL-6 and CINC-1 and with selective ET-A (BQ-123) and ET-B (BQ-788) endothelin receptors antagonists. The MT-II-induced hyperalgesia was not altered by the lipoxygenase inhibitor zileuton, by the bradykinin B_1_ receptor antagonist Lys-(Des-Arg9,Leu8)-bradykinin, by the histamine and serotonin antagonists promethazine and methysergide, respectively, by the nitric oxide inhibitor LNMMA and by the inhibitor of matrix 1-, 2-, 3-, 8- and 9- metalloproteinases GM6001 (Ilomastat).

**Conclusion:**

These results demonstrated the multi-mediated characteristic of the articular inflammation induced by MT-II, which demonstrates its relevance as a model for arthritis mechanisms and treatment evaluation.

## Background

Articular inflammations or arthritis are pathological conditions that affect around 54 million adults (23% of the population) only in USA [1]. Arthritis comprises more than 100 different diseases and conditions, being rheumatoid arthritis and osteoarthritis the two most common types. Other frequently occurring forms of arthritis include lupus and gout [2]. Rheumatoid arthritis and osteoarthritis are the most common inflammatory joint diseases, and their symptoms include aching, stiffness, and swelling in or around the joints, having pain and functional disability as their main consequences [2–4].

Articular inflammation is a multi-mediated condition, implying a role for mediators such as interleukin (IL)-1β, IL-6, tumour necrosis factor (TNF), platelet activating factor (PAF), and prostaglandin E_2_ (PGE_2_) [5]. Besides these and other mediators present in this pathology, the participation of phospholipases A_2_ (PLA_2_) in this process is also well documented [6].

The PLA_2_ superfamily includes 16 groups comprising six main types comprising the secreted (sPLA_2_), cytosolic (cPLA_2_), calcium-independent (iPLA_2_), platelet-activating factor acetylhydrolase (PAF-AH) also known as lipoprotein-associated (LpPLA_2_), lysosomal (LPLA_2_), and adipose (AdPLA) enzymes [7]. It has been demonstrated the presence of high levels of PLA_2_ in the synovial fluid of inflamed joints of animals and humans, being the PLA_2_activity increased in correlation with the severity of arthritis [8–11].

Many new therapies and strategies to control arthritis are currently being investigated, raising hopes for a better future for patients with this disease [12, 13]. In this context, experimental models that allow the study of the mechanisms underlying these inflammatory and pain conditions are of great clinical relevance.

PLA_2_s are widespread in nature, and can be found in a great diversity of tissues and fluids, including mammalian cells. These enzymes are notoriously abundant in venoms from snakes, bees, the *Heloderma* lizard, and the marine snail *Conodipina* sp [14–19]*.*

Four myotoxins with PLA_2_ structure have been isolated from the venom of the viperid snake *Bothrops asper*, named MT-I to MT-IV [20]. Despite high homology among these proteins, MT-II and MT-IV (which present a Lys instead of the canonical Asp residue at position 49) lack catalytic activity, whereas MT-I and MT-III (which contain an Asp residue at position 49) display high enzymatic activity [21, 22]. These PLA_2_s comprise approximately 30% of the venom proteins in this venom, and play a relevant role in its myotoxic, pro-inflammatory and hyperalgesic activities [18, 22, 23].

Regardless of their catalytic activity, both MT-II and III induce marked local inflammation and pain. Despite few differences in kinetics of release, both MT-II (Lys49-PLA_2_) and MT-III (Asp49-PLA_2_) are able to stimulate the production and release of inflammatory mediators such as IL-1 and IL-6, TNFα, LTB4, TXA_2_, PGE_2_ and PGD_2_ at the site of their injection as well as under in vitro conditions [24–27]. Concerning their hyperalgesic activity, both MT-II and MT-III cause significant local hyperalgesia in the rat hind paw after intraplantar injection, of rapid onset and similar time-course [28]. The mediators involved in the nociceptive process induced by both myotoxins are almost the same, differing in the level of the pain threshold [27–29]. These results indicate that enzymatic activity is not a strict requirement for the induction of nociception, but is important for determination of the intensity of the nociceptive phenomenon.

Therefore, the aim of this study was to establish a new model of myotoxin-induced joint acute arthritis to investigate the role of PLA_2_s in this process. For this purpose, MT-II was used because, not being itself catalytically-active, allows for the study of the phenomenon without the interference of exogenous enzymatic phospholipid degradation.

Our results demonstrated that in spite of its enzymatic inactivity, MT-II induces a multi-mediated acute articular inflammation that shares many of the features observed in humans arthritis. Thus, MT-II can be considered a suitable model for the determination of cellular and molecular mechanisms involved in arthritis process as well as a useful assay to evaluate new possible therapeutic compounds.

## Methods

### Isolation of Myotoxin II (MT-II)

MT-II, an enzymatically-inactive Lys49 PLA_2,_ was isolated from *Bothrops asper* venom obtained from adult specimens collected in the Caribbean region of Costa Rica, by ion-exchange chromatography on CM-Sephadex C-50, as previously described [30]. Salt-free, lyophilized MT-II was stored at −20 °C until use.

### Animals

Male Wistar rats (170–190 g) were used throughout this study. Animals were housed in a temperature-controlled (21 ± 2 ° C) and light-controlled (12/12 h light/dark cycle) room with standard food and water available ad libitum.

### Induction of articular inflammation

The articular inflammation was induced by administration of MT-II, in different doses, into the left tibio-tarsal or femoral-tibial-patellar joints, depending on the experimental protocol used, in rats lightly anesthetized by inhalation of halothane (Cristália Ltda, Brazil). MT-II was diluted in sterile PBS solution (NaCl 0.14 M; KCl 2.7 mM; Na_2_HPO_4_ 8.0 mM; KH_2_PO_4_ 1.5 mM) and injected in a volume of 25 or 50 μL into the tibio-tarsal or femoral-tibial-patellar joints, respectively, using an insulin syringe (0.5 mL, needle 5/16” 30G) inserted into the joint. For the femoral-tibial-patellar joint inflammation, carrageenin was used as positive control (200 μg/50 μL) and PBS (50 μL) was used as a control [31, 32]; while for the tibio-tarsal joint inflammation the control groups were constituted by animals that received zymosan (30 μg/ 25 μL, used as positive control) or bovine serum albumin (BSA, 20 μg/25 μL, used as a control of the protein content injected in the joint) or PBS (25 μL) [33–35].

### Determination of the cellular influx to the articulation

The cellular influx was evaluated using two methods.

#### Total and differential counts

To evaluate the cellular influx to the femoral**-**tibial**-**patellar articulation, the animals were terminally anaesthetized (halothane inhalation), killed by cervical dislocation and ex-sanguinated by sectioning the cervical vessels 1, 4, 8 and 12 h after MT-II (5, 10, 15 and 20 μg/joint) injection. The synovial cavity of the knee joints was then washed with 50 μL of PBS containing 4 mM of ethylenediaminetetraacetic acid. The synovial exudates were collected by aspiration and total and differential cell counts were performed using a Neubauer chamber (1:20 dilution v:v) and stained smears (violet crystal 0.5%), respectively. A total of 100 cells were counted on a light microscope.

#### Measurement of myeloperoxidase (MPO) activity

The tibio-tarsal joint region was separated from the tibio-tarsal bone complex at 8 h after MT-II (10 μg/joint) administration. The neutrophil migration to the tibio-tarsal joint region of rats was evaluated by the myeloperoxidase (MPO) kinetic-colorimetric assay as described previously [36]. Samples of joint tissue were collected and kept at −80 °C until use. Samples were placed in CTAB solution (hexadecyl trimethylammonium bromide 0.5%, prepared in 50 mM K_2_HPO_4_ buffer, pH 6.0) at 37 ° C, homogenized and centrifuged at 4,200 *g* for 10 min at 4 ° C. Briefly, 20 μL of the supernatant was mixed with 130 μL of ODP solution (*o*-Phenylene diamine, 10 mg, dissolved in 10 mL of phosphate buffer containing 1 μmol of hydrogen peroxide); and the mixture was assayed spectrophotometrically for MPO activity determination at 492 nm.

The determination of the cellular influx, assessed by the measurement of MPO activity was performed 8 h after intra-articular injection of MT-II (10 μg) or PBS, in animals pre-treated or not with fucoidan (5 mg/kg, i.v.), a sulfated polysaccharide that binds to L-selectin, 15 min prior to myotoxin.

### Trypan blue exclusion test of cell viability

Cell viability was determined using polymorphonuclear cells collected from peritoneal cavity by the Trypan blue exclusion method. Peritoneal cell migration was induced by i.p. injection of glycogen (10 mL). Four hours later, animals were euthanized in a CO_2_ chamber, ex-sanguinated by sectioning the cervical vessels and had the peritoneal cavity washed with 10 mL of cold PBS [37–39]. After gentle massage of the abdominal wall, the peritoneal fluid containing cells was collected. Cells were kept (1 × 10^6^ cells/mL) in RPMI 1640 medium with or without MT-II (5, 10, 15 and 20 μg/mL) for 1 h in a 37^o^ CO_2_ incubator. The dye exclusion counting was performed in a Neubauer’s hemocytometer using 1% Trypan blue. A total of 100 cells were counted by light microscopy.

### Plasma extravasation in the knee joint induced by myotoxin

The plasma extravasation was determined according to the protocol described by Lam and Ferrell [40]. Evans Blue dye (75 mg/kg) was injected i.v. 20 min before joint excision. MT-II was injected by intra-articular route and 5, 15, 30, 60, 240 and 360 min afterwards, animals were euthanized by cervical dislocation, exsanguinated by sectioning the cervical vessels and the knee joint capsules were dissected. These samples were weighed, cut into smaller pieces and mixed in a solution containing acetone and 1% aqueous solution of sodium sulphate (7:3 proportion). Samples were kept in continuous mild shaking for 24 h at room temperature. Each preparation was then centrifuged at 2000 rpm for 10 min. The supernatant was collected and the amount of dye recovered was calculated by comparing the absorbance of the supernatant at 620 nm (Labsystems MuItiscan) with that of a standard curve prepared with known concentrations of Evans blue.

As Evans blue dye binds to plasma proteins normally restricted to the vascular compartment, its presence in the capsule provides an index of altered vascular permeability. In this experiment, the control group was constituted of animals that received Ringer-Lock solution injected by intra-articular route. The amount of tissue obtained from each animal was small, thus requiring the pooling of the samples. Then, for each experimental procedure, four groups of three rats were used. Results are expressed as μg Evans blue/mL.

### Evaluation of edema

The edematogenic response induced by myotoxin was evaluated in both tibio-tarsal and femoral-tibial**-**patellar joints. MT-II (10 g/articulation) was diluted in 25 (tibio-tarsal articulation) or 50 μL (femoral-tibial**-**patellar articulation) of PBS. The same volume of PBS was injected in the contralateral articulation. The increase in the articulation was determined by measuring joint thickness using a caliper at 0 (time before injections), 1, 2, 4, 8 and 24 h after MT-II or PBS injection. Results were calculated by the difference in thickness of both joints, and edema was expressed as the percentage increase in joint thickness as compared to the control.

### Evaluation of articular hypernociception

The articular hypernociception was determined by a dorsal flexion of the tibio-tarsal joint, evaluated using a modified electronic pressure-meter test, as previously described [34]. Rats were placed in acrylic cages with a wire grid floor 20 min before testing for environmental adaptation. A tilted mirror was placed below the grid floor to provide a clear view of the hind paw. Stimulations were performed only when animals were quiet, did not display exploratory movements or defecation, and were not resting on their paws. In these experiments, an electronic pressure meter was used. It consists of a hand-held force transducer fitted with a polypropylene tip (Insight Ltda, Brazil) with a large tip (4.15 mm^2^) adapted to the probe.

In this test, an increasing perpendicular force is applied to the central area of the plantar surface of the hind paw to induce flexion of the tibio-tarsal joint, and this force is automatically interrupted when the animal reacts by withdrawing the paw. The electronic pressure-meter apparatus automatically recorded the intensity of the force necessary to induce this animal reaction. The test was repeated until three measurements with less than 1 g of variation were obtained. The flexion-elicited mechanical threshold was expressed in grams (g). The test was applied before and in different times after the intra-articular injection of MT-II (10 μg) or BSA (20 μg), zymosan (30 μg) and PBS, used as controls.

### Pharmacological treatments

In order to investigate the mechanisms involved in the articular hypernociception induced by MT-II, receptor antagonists and enzymatic inhibitors were used:

- To evaluate the contribution of the cellular influx to the joint to the hypernociceptive effect, fucoidan (5 mg/kg, i.v.), a sulfated polysaccharide that binds to L-selectin, was injected 15 min prior to MT-II [41].
- To investigate the involvement of arachidonate metabolites in this phenomenon, different groups of rats were treated with the cyclooxygenase inhibitor indomethacin (4 mg/kg, 30 min before myotoxin), with the type-2 cyclooxygenase inhibitor celecoxib (10 mg/kg, 60 min before myotoxin) or with the 5-lipoxygenase inhibitor zileuton (100 mg/kg, 60 min before myotoxin) [28, 42].
- In order to assess the involvement of endogenous PLA_2_ activity to the myotoxin-induced hypernociception, rats were treated with arachidonyl trifluoromethil ketone (AACOCF_3_, 200 μg/joint), a potent and selective inhibitor of cPLA_2_, or palmitoyl trifluoromethyl ketone (PACOCF_3_, 1 μg/joint), an inhibitor of iPLA_2_, 30 min before myotoxin administration [43, 44].
- To evaluate the participation of bradykinin in the algogenic effect of myotoxin, a bradykinin B_1_ receptor antagonist Lys-(Des-Arg^9^,Leu^8^)-bradykinin (Lys-BK, 10 and 40 nmol) and a bradykinin B_2_ receptor antagonist icatibant (HOE 140, 0.75 μmol) were injected by the intra-articular route 20 min before myotoxin administration [28, 45].
- To evaluate the contribution of cytokines, animals were treated with an anti-TNF-α antibody (0.5 μg/joint), with an anti-interleukin-1β antibody (1.5 μg/joint), with an anti-interleukin-6 antibody (4.0 μg/joint) or with an anti-CINC-1 antibody (5.0 μg/joint), 30 min before myotoxin. Carragenin (200 μg/joint) was used as positive control of the antibody doses used since carragenin-induced hypernociception is abrogated by these antibodies.
- To examine the participation of histamine and serotonin, animals were injected with promethazine or methysergide (5 mg/kg, i.p.) 30 min before myotoxin injection [28].
- To explore the effect of endothelin, BQ-123 and BQ-788 (10 and 20 nmol/joint), selective antagonists of ET-A and ET-B endothelin receptors, were injected 30 min before myotoxin administration [46].
- In order to investigate the participation of metalloproteinases in the MT-II effects, Ilomastat (GM6001, 27 and 71 nM/joint), a potent broad-spectrum hydroxamate inhibitor of matrix metalloproteinases (inhibitor of 1-, 2-, 3-, 8- and 9-MMPs) was injected 30 min before myotoxin administration. Zymosan (30 μg/joint) was used as positive control of GM6001 doses since it is capable of increasing the mRNA expression to MMPs-2, −3 and −9 in the synovial tissue [47].
- In order to investigate the participation of nitric oxide (NO) on myotoxin-induced hypernociception, rats were treated with the inhibitor of nitric oxide synthase (NOS), _L_-NMMA (50 μg/joint), 60 min before myotoxin injection [48].

Indomethacin was diluted in Tris buffer (1 M, pH 8.0 at 37^o^ C) and PBS. Celecoxib and zileuton were dissolved in CMC 1%. HOE 140, Lys-(Des-Arg9,Leu8)-bradykinin, anti-IL-1β, anti-IL-6, anti-TNFα and anti-CINC-1 antibodies were diluted in PBS. BQ-123 and BQ-788 were diluted in distilled water. GM6001, AACOCF_3_ and PACOCF_3_ were dissolved in DMSO. LNMMA, promethazine, methysergide and fucoidan were diluted in saline. In all experiments, control groups were constituted of animals treated with MT-II plus the specific diluents of each drug.

#### Drugs used

Anti-IL-1β, anti-IL-6, anti-TNFα and anti-CINC-1 antibodies were supplied by R&D Systems Inc. (USA). Indomethacin, AACOCF_3_ and PACOCF_3_ were purchased from Biomol Research Laboratories (USA). GM6001 was supplied by USBiological (USA); whereas L-NMMA, HOE 140, Lys-(Des-Arg9,Leu8)-bradykinin, promethazine, methysergide, BQ-123, BQ-788 and fucoidan were purchased from Sigma-Aldrich Co. (USA). Celecoxib was supplied by Searle and Co (Puerto Rico). Zileuton was purchased from Abbott Laboratories (Zyflo®, USA). Carrageenin was purchased from Marine Colloids.

#### Statistical analysis

Results are presented as mean ± S.E.M. Statistical evaluation of data was carried out by analysis of variance (ANOVA) and sequential differences among means were compared according to Tukey contrast analysis at *p <* 0.05 [49].

## Results

### Cellular migration induced by myotoxin II

An increase in the total influx of cells into the femoral-tibial-patellar joints of animals was noticed 8 h after intra-articular injection of myotoxin, only with the dose of 10 μg/joint. This increase was comparable to the cell influx induced by carrageenin, used as positive control, and is due to an increase in the numbers of polymorphonuclear cells (Table 1). When animals were treated with other doses of myotoxin (5, 15 and 20 μg/joint) or BSA, used as a control of the quantity of protein injected in the joint, no statistically significant difference was noted for cell migration values when compared to groups treated with PBS (Table 1).

**Table 1:** Myotoxin-induced cell migration to the joint

Treatment	Groups (*n =* 6)	Total cells (× 10^6^/mL)				Mononuclear cells (× 10^6^/mL)	PMN cells (× 10^6^/mL)
		1 h	4 h	8 h	12 h	8 h	8 h
PBS	6	2.12 ± 0.70	3.72 ± 1.80	1.09 ± 0.61	0.24 ± 0.14	0.25 ± 0.13	1.65 ± 0.77
Myotoxin 5 μg/joint	6	2.07 ± 0.6	83.44 ± 25.2	52.27 ± 23.67	5.20 ± 1.54	7.97 ± 4.65	44.30 ± 19.07
Myotoxin 10 μg/joint	6	18.71 ± 4.10	73.16 ± 22.10	163.08 ± 48.04^a^	28.12 ± 7.57	8.15 ± 2.87	154.93 ± 34.16^a^
Myotoxin 15 μg/joint	6	6.12 ± 1.80	48.5 ± 22.70	66.38 ± 21.50	21.32 ± 8.38	3.91 ± 0.86	62.47 ± 13.73
Myotoxin 20 μg/joint	6	10.90 ± 12.75	41.37 ± 20.10	43.15 ± 5.53	12.75 ± 3.40	2.79 ± 0.85	40.36 ± 4.88
Carrageenin	6	4.74 ± 1.10	49.43 ± 7.20	109.14 ± 30.31^a^	15.64 ± 3.65	7.21 ± 2.36	101.93 ± 28.22^a^
BSA	6	0.50 ± 0.30	1.00 ± 0.5	1.50 ± 3.40	2.00 ± 0.50	/	/

Total and differential cellular influx to the femoral-tibial-patellar articulation, evaluated 1, 4, 8 and 12 h after myotoxin II (5, 10, 15 and 20 μg/joint) injection. Total and differential cell counts were performed using a Neubauer chamber (1:20 dilution v:v) and stained smears (violet crystal 0.5%), respectively. A total of 100 cells were counted on a light microscope

^a^Significantly different from mean values of control group (BSA)

### Trypan blue exclusion test of cell viability

Since the increase in the cell influx was observed just for the dose of 10 μg/joint of myotoxin, we used the dye exclusion test to determine the number of viable cells collected from peritoneal cavity after treatment with MT-II.

After 1 h exposure, trypan blue exclusion assay revealed that cell viability of groups treated with PBS, 5 μg of myotoxin and 10 μg of myotoxin was 100%, while in the groups treated with 15 and 20 μg the cell viability was 50 and 20%, respectively. Based on these findings, and in agreement with the results obtained in the cellular migration assay, the dose of 10 μg/joint of myotoxin/joint was chosen for subsequent tests.

### Plasma extravasation in the knee joint induced by myotoxin

Plasma extravasation in the knee joints was determined 5, 15, 30, 60, 240 and 360 min after myotoxin injection. Results demonstrated an increase of 25 and 57% in the concentrations of Evans blue dye in the samples from animal treated with myotoxin 5 and 15 min after injection, respectively, when compared with animal treated with Ringer-Lock solution. No statistically significant difference was noted for plasma extravasation values in the subsequent times.

### Characterization of articular hypernociception and edema

**Fig. 1:**
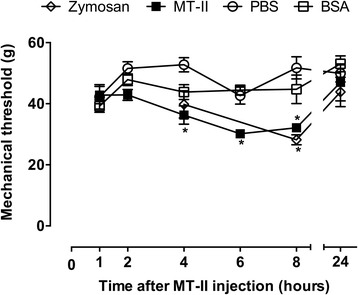
MT-II-induced articular hyperalgesia. MT-II (10 μg/joint) or PBS (vehicle) was injected in tibio-tarsal articulation (25 μL). Pain threshold was determined by a dorsal flexion of the tibio-tarsal joint using a modified electronic pressure-meter test before (time 0 – basal) and 1, 2, 4, 6, 8 e 24 h after MT-II injection, and was represented as force (in *g*). Zymosan (30 μg) and BSA (20 μg) were used as controls. Each point represents the mean ± SEM of six animals. *p < 0.05 indicate statistically significant differences when compared with PBS group (vehicle)

The intraplantar injection of myotoxin II (10 μg/joint) into the rat tibio-tarsal joint caused a significant decrease in pain threshold (Fig. 1). The hypernociception was detected from 4 to 8 h, decreasing thereafter and completely disappearing within 24 h. Zymosan (30 μg/joint) used as positive control, induced hypernociception with same intensity of myotoxin, observed 8 h after its injection (Fig. 1) [34]. The injection of saline or BSA (control groups) did not modify the pain threshold of the animals (Fig. 1).

**Fig. 2:**
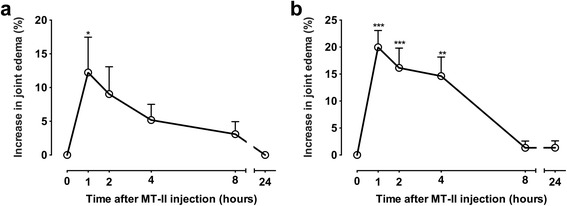
Edema induced by MT-II in (**a**) tibio-tarsal and (**b**) femoral-tibial-patellar rat joints. MT-II (10 μg/articulation) was injected in 25 (tibio-tarsal articulation) or 50 μL (femoral**-**tibial-patellar articulation) of PBS (vehicle). The same volume of PBS was injected in the contralateral articulation. The increase in the articulation was determined by measuring the joint edema using a caliper at 0 (time before injections) or 1, 2, 4, 8 and 24 h after MT-II or PBS injection. Results are expressed as the percentage in the increase in joint thickness of MT-II group in relation to the PBS group. Each point represents the mean ± SEM of six animals. *p < 0.05, **p < 0.01 and ***p < 0.001 indicate statistically significant differences when compared with baseline (time 0)

In agreement, the injection of myotoxin caused a time-dependent edema, observed in both tibio-tarsal (Fig. 2a) and femoral-tibial-patellar (Fig. 2b) joints. In both joints, the maximum increase in hind-paw swelling occurred 1 h after MT-II injection, decreasing thereafter and completely disappearing within 24 h (Fig. 2).

### Contribution of the cellular influx to the joint to the hypernociceptive effect of myotoxin

**Fig. 3:**
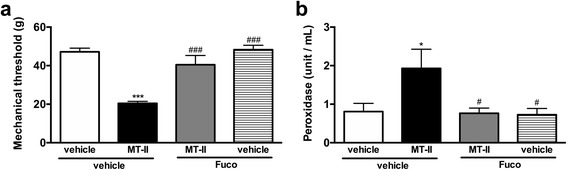
Effect of a L-selectin binder on MT-II- induced articular hyperalgesia. MT-II (10 μg/joint) or PBS (vehicle) was injected in tibio-tarsal articulation (25 μL). Fucoidan (fuco), a L-selectin binder (5 mg/kg, i.v.) or saline (vehicle) was injected 15 min prior to MT-II. **a** Pain threshold was determined using a modified electronic pressure-meter test 8 h after MT-II injection, and represented as force (in *g*). **b** The neutrophil migration to the tibio-tarsal joint region of mice was evaluated by the myeloperoxidase (MPO) kinetic-colorimetric assay, tested 8 h after MT-II injection. Each point represents the mean ± SEM of six animals. *p < 0.05 and ***p < 0.001 indicate statistically significant differences when compared with control group (vehicle + vehicle). #*p* < 0.05 and ###*p* < 0.001 indicate statistically significant differences when compared with MT-II group (MT-II + vehicle)

The treatment with fucoidan, a sulfated polysaccharide that binds to L-selectin, prevented the hyperalgesia induced by myotoxin (Fig. 3a). The efficacy of fucoidan in decrease the cellular influx to the joint was confirmed in the MPO activity assay (Fig. 3b).

### Mediation of myotoxin-induced hypernociceptive effect

#### Participation of eicosanoids and endogenous phospholipases A_2_

**Fig. 4:**
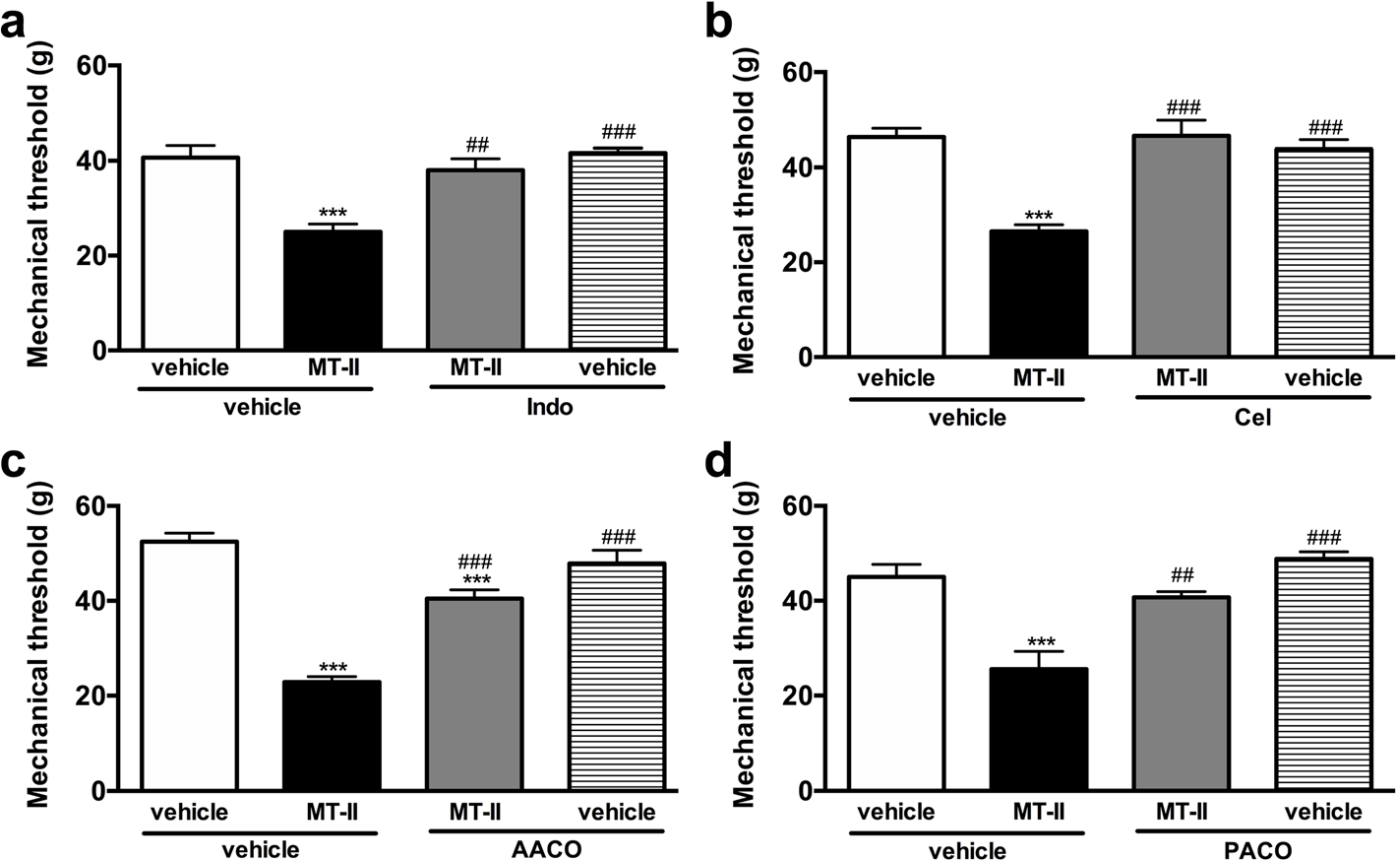
Involvement of eicosanoids and endogenous phospholipases A_2_ on MT-II- induced articular hyperalgesia. MT-II (10 μg/joint) or PBS (vehicle) was injected in tibio-tarsal articulation (25 μL). Pain threshold was determined using a modified electronic pressure-meter test 8 h after MT-II injection, and represented as force (in *g*). **a** Indomethacin, a cyclooxygenase inhibitor (Indo, 4 mg/kg, 30 min before MT-II) or (**b**) celecoxib, a type-2 cyclooxygenase inhibitor (Cel, 10 mg/kg, 60 min before MT-II) or (**c**) arachidonyl trifluoromethil ketone, a selective inhibitor of cPLA_2_ (AACO, 200 μg/joint, 30 min before MT-II) or (**d**) palmitoyl trifluoromethyl ketone, an inhibitor of iPLA_2_ (PACO, 1 μg/joint, 30 min before MT-II) was injected prior to MT-II. Each point represents the mean ± SEM of six animals. ***p < 0.001 indicate statistically significant differences when compared with control group (vehicle + vehicle). ##*p* < 0.01 and ###*p* < 0.001 indicate statistically significant differences when compared with MT-II group (MT-II + vehicle)

Pretreatment with the cyclooxygenase inhibitor indomethacin (Fig. 4a) or type 2 cyclooxygenase inhibitor celecoxib (Fig. 4b) significantly reduced the hyperalgesia caused by myotoxin. The lipoxygenase inhibitor zileuton did not modify the hyperalgesic response (Table 2).

**Table 2:** Evaluation of histamine, serotonin, nitric oxide and metalloproteinases in the myotoxin-induced hypernociceptive effect

Treatment	Force in grams evaluated 8 h after myotoxin injection
Saline + PBS	49.90 ± 1.72
DMSO + PBS	49.60 ± 2.11
Saline + Myotoxin	25.58 ± 0.59^a^
DMSO + Myotoxin	23.64 ± 0.79^a/NS^
Zyleuton + Myotoxin	29.82 ± 3.03^a/NS^
Methysergide + Myotoxin	26.72 ± 0.85^a/NS^
Promethazine + Myotoxin	24.74 ± 1.04^a/NS^
L-NMMA + Myotoxin	29.14 ± 1.72^a/NS^
GM6001 + Myotoxin	26.06 ± 0.69^a/NS^
Methysergide + PBS	49.82 ± 1.31
Promethazine + PBS	50.06 ± 2.18
L-NMMA + PBS	48.26 ± 3.87
GM6001 + PBS	44.54 ± 2.33

Articular hyperalgesia induced by MT-II in rats in the presence or in the absence of different pharmacological treatments. The articular hypernociception was determined by a dorsal flexion of the tibio-tarsal joint using a modified electronic pressure-meter test and was represented as force (in *g*), observed 8 h after MT-II injection. Zyleuton: 5-lipoxygenase inhibitor; methysergide: antagonist of H_1_ histaminergic receptor; promethazine: antagonist of serotoninergic receptors; L-NMMA: inhibitor of nitric oxide synthase; GM6001: a potent broad-spectrum hydroxamate inhibitor of matrix metalloproteinases (inhibitor of 1-, 2-, 3-, 8- and 9-MMPs)

*NS* Not significantly different from mean values of myotoxin group

^a^Significantly different from mean values of control group (Saline or DMSO + PBS)

Since it was demonstrated that both cyclooxygenase and type 2 cyclooxygenase inhibitors blocked the hyperalgesic effect of myotoxin and considering that this myotoxin is an enzymatically-inactive PLA_2_, we investigated the possible participation of endogenous phospholipases in this effect, since myotoxin cannot hydrolyze membrane phospholipids directly.

Results demonstrated the both AACOCF_3_ (Fig. 4c) and PACOCF_3_ (Fig. 4d) prevented the hypernociception induced by myotoxin, suggesting the participation of cytosolic and Ca^2+^-independent PLA_2_s in this effect.

#### Participation of bradykinin

**Fig. 5:**
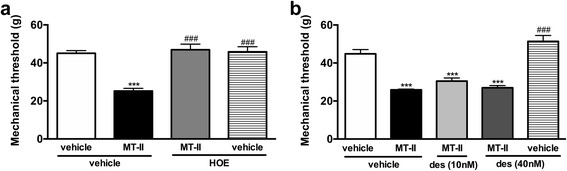
Involvement of bradykinin on MT-II-induced articular hyperalgesia. MT-II (10 μg/joint) or PBS (vehicle) was injected in tibio-tarsal articulation (25 μL). Pain threshold was determined using a modified electronic pressure-meter test 8 h after MT-II injection, and represented as force (in *g*). (**a**) A bradykinin B_2_ receptor antagonist icatibant (HOE 140, 0.75 μmol) or (**b**) a bradykinin B_1_ receptor antagonist Lys-(Des-Arg^9^,Leu^8^)-bradykinin (des, 10 and 40 nmol) was injected by intra-articular route 20 min prior to MT-II. Each point represents the mean ± SEM of six animals. ***p < 0.001 indicate statistically significant differences when compared with control group (vehicle + vehicle). ###*p* < 0.001 indicate statistically significant differences when compared with MT-II group (MT-II + vehicle)

Myotoxin-induced hyperalgesia was abolished by treating the animals with the bradykinin B_2_ receptor antagonist HOE 140 (Fig. 5a), but it was not altered by bradykinin B_1_ receptor antagonist Lys-(Des-Arg9,Leu8)-bradykinin (Fig. 5b).

#### Participation of cytokines

**Fig. 6:**
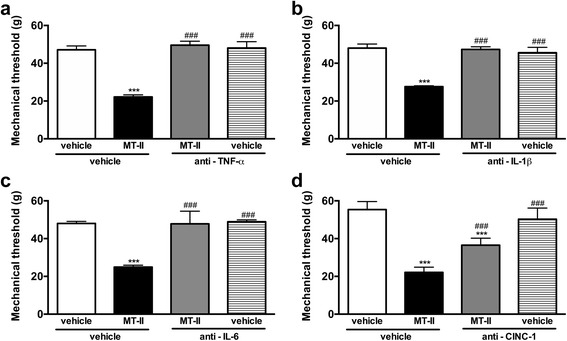
Involvement of cytokines on MT-II-induced articular hyperalgesia. MT-II (10 μg/joint) or PBS (vehicle) was injected in tibio-tarsal articulation (25 μL). Pain threshold was determined using a modified electronic pressure-meter test 8 h after MT-II injection, and represented as force (in *g*). **a** anti-TNFα antibody (0.5 μg/joint) or (**b**) anti-IL-1β antibody (1.5 μg/joint) or (**c**) anti-IL-6 antibody (4.0 μg/joint) or (**d**) anti-CINC-1 antibody (5.0 μg/joint) was injected 30 min before MT-II. Each point represents the mean ± SEM of six animals. *** *p* < 0.001 indicate statistically significant differences when compared with control group (vehicle + vehicle). ### *p* < 0.001 indicate statistically significant differences when compared with MT-II group (MT-II + vehicle)

Pretreatment with antibodies against TNFα (Fig. 6a), IL-1β (Fig. 6b) and IL-6 (Fig. 6c) blocked the hypernociceptive effect of myotoxin. Antibodies against CINC-1 partially reduced this effect (Fig. 6d).

#### Participation of endothelin

**Fig. 7:**
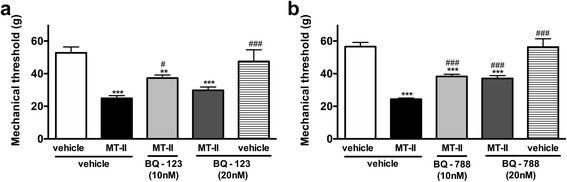
Involvement of endothelin on MT-II-induced articular hyperalgesia. MT-II (10 μg/joint) or PBS (vehicle) was injected in tibio-tarsal articulation (25 μL). Pain threshold was determined using a modified electronic pressure-meter test 8 h after MT-II injection, and represented as force (in *g*). **a** BQ-123 or (**b**) BQ-788 (10 and 20 nmol/joint, selective antagonists of ET-A and ET-B endothelin receptors, respectively) were injected 30 min before MT-II. Each point represents the mean ± SEM of six animals. **p < 0.01 and ***p < 0.001 indicate statistically significant differences when compared with control group (vehicle + vehicle). #*p* <0.05 and ### *p* < 0.001 indicate statistically significant differences when compared with MT-II group (MT-II + vehicle)

The hypernociceptive effect induced by myotoxin was partially reversed by the pretreatment with BQ-123 and BQ-788, selective antagonists of ET-A (Fig. 7a) and ET-B (Fig. 7b) endothelin receptors respectively.

#### Participation of histamine, serotonin, nitric oxide and metalloproteinases

The histamine and serotonin antagonists promethazine and methysergide, respectively, the nitric oxide inhibitor LNMMA and the inhibitor of matrix metalloproteinases 1-, 2-, 3-, 8- and 9- GM6001 (Ilomastat) did not interfere with the effect of myotoxin (Table 2).

## Discussion

Although outstanding efforts have been performed by clinicians and researchers to find effective strategies to treat and to restore joint function in articular inflammatory conditions such as osteoarthritis and rheumatoid arthritis, effective and/or protective treatments are still a challenge [50, 51]. For this reason, animal models that share the same characteristics of human arthritis are useful for the characterization of these conditions and for the study of new therapies.

The multi-mediated characteristic of articular inflammatory diseases and the importance of PLA_2_s and cyclooxygenase-derived mediators to these conditions have been well documented [5, 6]. Interestingly, studies performed using MT-II, a catalytically inactive PLA_2_ homologue, demonstrated that its enzymatic activity is not essential for its proinflammatory effects, since it is able to induce eicosanoid production through the stimulation of endogenous cytosolic and Ca^2+^-independent phospholipases A_2_ [26, 52, 53]. Herein it was demonstrated that in spite of lacking enzymatic activity, MT-II can induce acute arthritis, allowing the study of mediators involved in this condition. Our observations indicate that this is a multi-mediated process that involves the participation of eicosanoids (through the activation of endogenous PLA_2_s), bradykinin, cytokines, endothelin and is dependent on the cellular influx to the joint.

Both MT-II (Lys49-PLA_2_) and MT-III (Asp49-PLA_2_) induce hyperalgesia, allodynia, edema, plasma extravasation and H_2_O_2_ production by isolated macrophages [24, 28, 29, 54, 55]. The difference among the myotoxins is the intensity of their effects, since in all of them the effect observed with MT-II is weaker than that of MT-III. The Lys49-PLA_2_ was chosen for the present study since the lack of enzymatic activity eliminates the possibility that exogenous PLA_2_ degradation of phospholipids may contribute to the genesis of the inflammation, thus allowing the study of the role of endogenous, inflammatory PLA_2_s in this phenomenon.

The kinetics of the articular inflammation induced by MT-II was characterized. MT-II induced a rapid plasma extravasation in the knee joints observed 5 min after its injection, which peaked at 15 min. A time-dependent edema was observed in both tibio-tarsal and femoral-tibial-patellar joints, reaching its maximum increase 1 h after myotoxin injection. The inflammatory response reached its peak 8 h after MT-II injection, a time when the cell influx and hyperalgesic effect reached their maximum. In these studies, the selected dose (10 μg) was not cytotoxic. Previous studies already demonstrated that MT-II induces prominent leukocyte infiltration to the peritoneal cavity 6 h after its injection, composed predominantly of polymorphonuclear leukocytes [24]. This same cell migration profile was obtained in the present study using carrageenan and is in agreement with previous studies [56], confirming articular MT-II injection as a suitable model for articular inflammation evaluation.

According to the World Health Organization rheumatoid arthritis and osteoarthritis are included in the group of conditions having the greatest impact on society, being osteoarthritis one of the ten most disabling diseases in developed countries [57]. In addition, pain can be considered one of the most prominent symptoms in people suffering of arthritis, being the most important cause of disability and loss of joint function in patients with osteoarthritis [57, 58]. Considering this, the hyperalgesic effect of articularly injected MT-II was investigated and the role of several inflammatory mediators in this process was determined.

MT-II induced significant hyperalgesia which peaked 8 h after injections. The hyperalgesic effect of both MT-II and MT-III was previously investigated after intraplantar injection of the toxins [28]. These authors demonstrated that MT-II induced hyperalgesia that peaked 1 h later after intraplantar injection, decreasing afterwards. Differences in the experimental conditions between that study and our present report, particularly regarding the site of injection, could explain the differences described. In our case, it is interesting to note that the peak of the hyperalgesic response of the animals coincided with the peak of cell influx.

The cellular traffic between the blood and the tissues is regulated by adhesion molecules expressed on the blood and endothelial cell surface [59]. Among the major adhesion molecules involved in cell transmigration is L-selectin, a molecule indispensable for adhesion, diapedesis and subsequent cell migration to the tissue [60, 61]. Thus, the importance of cell influx to the hyperalgesic effect induced by MT-II was investigated using fucoidan, a binder of L-selectin which is able to inhibit cell migration into the tissue in a dose that does not affect the number of circulating leukocytes [41]. Our data showing that fucoidan fully reverted the hyperalgesia induced by MT-II confirmed the importance of cell influx to the joint to MT-II-induced hyperalgesia. The reduction in cell migration into the joint cavity was confirmed by myeloperoxidase assay.

It is important to point out that previous studies demonstrated that fucoidan significantly inhibited both cytotoxic and myotoxic effects of MT-II and that this inhibition is due to a rapid formation of complexes between fucoidan and myotoxins [62]. Regardless this interference of fucoidan in MT-II-induced myotoxicity, it probably does not explain the inhibition of MT-II-induced hyperalgesia observed in our results, because this interference was observed only when fucoidan was incubated with MT-II or when they were injected simultaneously at the same site [62, 63]. In contrast, MT-II-induced muscle necrosis was not inhibited when fucoidan was administered by i.v. route, immediately after i.m. toxin injection [63]. Therefore, considering that in our studies fucoidan was administered by i.v. route and MT-II directly in the joint, it is possible to consider that the inhibition of MT-II-induced hyperalgesia was a consequence of the decrease in leukocytes migration into joint articulation.

This hyperalgesic effect clearly involves the participation of type 2 cyclo-oxygenase-derived mediators, since both indomethacin and celecoxib inhibited this effect. The lipoxygenase inhibitor zileuton did not modify the hyperalgesic response, suggesting that leukotrienes are not likely to be involved in this phenomenon. These results are in agreement with Chacur et al. [28], who had previously demonstrated the involvement of prostaglandins and the absence of leukotrienes on MT-II-induced hyperalgesia using the intraplantar injection model. Considering that MT-II is a PLA_2_-like protein devoid of catalytic activity and, therefore, cannot hydrolyze membrane phospholipids directly, the participation of cytosolic and Ca^2+^-independent endogenous PLA_2_s was presently investigated.

The combined activities of sPLA_2_ and endogenous cPLA_2_ or Ca^2+^-independent PLA_2_ to induce eicosanoid formation in different cells has already been proposed [64, 65]. In addition, previous works have demonstrated the ability of MT-II to induce inflammation through endogenous PLA_2_s activation. Moreira et al. [26] demonstrated that MT-II is able to induce PGD_2_ and PGE_2_ release and expression of COX-2 in macrophages in culture, being these phenomena decreased by the inhibition of cytosolic PLA_2_ but not Ca^2+^ independent PLA_2_. Giannotti et al. [52], investigated the ability of MT-II to induce, in isolated macrophages, the formation of lipid droplets (LD), which are key elements of inflammatory responses. It was demonstrated that iPLA_2_, but not cPLA_2_, signaling pathways are involved in this LD formation. Corroborating these data, our results showed that, in the joint, both cytosolic and Ca^2+^-independent phospholipases are involved in MT-II-induced articular hyperalgesia.

The role of several mediators on MT-II PLA_2_-induced hyperalgesia was presently investigated using inhibitors of specific pathways or receptor antagonists. It was observed that this effect involves the participation of bradykinin, acting through B_2_ receptors, indicating the importance of kinins to the hyperalgesic effect. Bradykinin is an inflammatory mediator involved in both pain and nociceptor sensitization [66, 67]. It was already demonstrated that in some inflammatory conditions, bradykinin may induce the release of several mediators that act in a cascade fashion, causing both pain and nociceptors sensitization. These are considered multi-mediated processes that involve participation of biogenic amines, cytokines (TNFα, IL-6, IL-1β and IL-8), prostanoids and sympathomimetic amines [66, 68–72]

The importance of bradykinin to the onset of pain in articular inflammatory conditions has also been highlighted. Severe acute pain is considered the most important clinical symptom in patients suffering from crystal-induced arthritis (CIA). Ramonda et al. [73], evaluating this phenomenon, demonstrated that bradykinin can be included as one of the most important molecules to induce pain, together with prostaglandins, cytokines (in particular, interleukin-1β) and substance P, exerting their effects through different receptors present in both peripheral sensory neurons and in the spinal cord. De Falco et al. [74] reviewed the importance of bradykinin to osteoarthritis and described the action of B_2_ receptor antagonists to this condition, presenting these antagonists as promising agents to the osteoarthritis treatment.

In spite of the fact that (i) bradykinin-induced pain partly depends on the release of inflammatory mediators by mast cells [75]; (ii) the release of vasoactive amines from mast cells incubated with venom cationic PLA_2_s has been previously detected [76, 77] and (iii) Chacur et al. [28] demonstrated that the hyperalgesic effect of MT-II injected in the rat paw is partially mediated by histamine and serotonin; these mediators do not seem to be involved in the MT-II-induced articular hyperalgesia, since both histamine and serotonin antagonists did not interfere with the hyperalgesic effect of MT-II. In addition, nitric oxide inhibitor LNMMA and the inhibitor of matrix 1-, 2-, 3-, 8- and 9-metalloproteinases GM6001 (Ilomastat) did not interfere with the effect of myotoxin. Although the importance of these mediators to inflammatory conditions is well stablished, it is suggested that they are not contributing to the observed hyperalgesic effect [47, 78–81].

The role of cytokines in hyperalgesic and inflammatory processes, including arthritis, is well documented [82–84]. The sensitization of nociceptors by cytokines is a multi-mediated process that involves the release of prostaglandins and sympathomimetic amines [68, 69, 72, 85, 86]. In addition, the release of cytokines induced by both *Bothrops asper* venom or isolated Lys49 PLA_2_ has already been described [28, 29, 55, 87, 88]. In agreement with these data, our results confirmed the importance of cytokines to the articular inflammation induced by MT-II, since antibodies against TNFα, IL-1β, IL-6 and CINC-1 interfered with the effects induced by MT-II.

Endothelins are peptides implicated in pain transmission in both humans and animals, which contribute to sensory changes associated with inflammatory and neuropathic pain [89–91]. In addition, these peptides have been involved in articular inflammatory conditions, including osteoarthritis, where endothelin signaling may play a role in destruction of bone-cartilage unit [92]. Thus, the participation of endothelin acting on ET-A or ET-B receptors in MT-II induced articular pain was investigated. Our results demonstrated that both ET-A and ET-B antagonists partially reversed the hyperalgesic effect of MT-II, even when both antagonists were associated (data not shown). These results underscore the involvement of endothelin in the MT-II-induced pain and suggest that the mediators involved in this pain signaling are not released in a sequencial manner, but probably through parallel pathways.

## Conclusion

In conclusion, our work demonstrated that MT-II, a catalytically-inactive Lys49-PLA_2_, induces an acute multi-mediated inflammatory articular process that includes most of the important mediators described in articular chronic conditions. Considering that arthritis is a pathological condition that has no cure, more in vivo animal models and clinical studies are needed to better understand the cellular and molecular mechanisms involved in this process as well as the efficacy and tolerability of new therapeutic compounds. In this context, MT-II-induced articular inflammation can be considered a valuable model for arthritis pathology and treatment evaluation.

## Abbreviations

CIA: Crystal-induced arthritis
cPLA_2_: Cytosolic phospholipase A_2_
i.m.: Intramuscular
i.p.: Intraperitoneal
i.v.: Intravenous
IL: Interleukin
iPLA_2_: Calcium-independent phospholipase A_2_
LD: Lipid droplets
MPO: Myeloperoxidase
MT-II: Myotoxin II
PAF: Platelet activating factor
PAF-AH: Platelet-activating factor acetylhydrolase
PGE_2_: Prostaglandin E_2_
PLA_2_: Phospholipase A_2_
sPLA_2_: Secreted phospholipase A_2_
TNF: Tumour necrosis factor
